# Stem Cell Secretome Treatment Reduces Adiposity and Improves Glucose Handling During Obesity and Weight Loss in Mice

**DOI:** 10.1002/oby.70068

**Published:** 2025-10-27

**Authors:** Zachary J. Fennel, Anu S. Kurian, Paul‐Emile Bourrant, Chad M. Skiles, Robert J. Castro, Elena M. Yee, Scott A. Greilach, Hans S. Keirstead, Gabriel Nistor, Nicole C. Berchtold, Thomas E. Lane, Micah J. Drummond

**Affiliations:** ^1^ Diabetes & Metabolism Research Center University of Utah Salt Lake City Utah USA; ^2^ Department of Physical Therapy and Athletic Training University of Utah Salt Lake City Utah USA; ^3^ Molecular Medicine Program University of Utah Salt Lake City Utah USA; ^4^ Department of Nutrition & Integrative Physiology University of Utah Salt Lake City Utah USA; ^5^ Immunis, Inc. Irvine California USA; ^6^ Department of Neurobiology and Behavior University of California Irvine California USA

**Keywords:** metabolism, obesity, stem cells, weight loss

## Abstract

**Objective:**

In this study, we investigated the effects of a stem cell‐derived secretome product on adiposity and tissue quality and insulin and glucose levels in obese mice and those undergoing dietary weight loss.

**Methods:**

Following 16 weeks of high fat diet mice received acute (4 weeks) biweekly intramuscular injections with vehicle or secretome while remaining on a high fat diet (HFD vs. HFD‐S) or during weight loss upon return to a normal chow diet (HFD/WL vs. HFD/WL‐S).

**Results:**

After 4 weeks of treatment, HFD‐S mice had greater lean mass (vs HFD), muscle weights, and quadriceps myofibrillar size and improved muscle quality (capillary density and fibrosis). HFD/WL‐S mice had accelerated whole‐body fat loss and improved glucose handling, fasting insulin levels, and HOMA‐IR. In both secretome‐treated groups (HFD‐S and HFD/WL‐S), liver steatosis and fibrosis were improved and similar to chow controls.

**Conclusions:**

Together, these results support that a stem cell secretome treatment may be useful to improve tissue quality and metabolic health during obesity and weight loss.

**Study Importance**

- What is already known?
  ○ Intramuscular treatment with a stem cell secretome product improves body composition and whole‐body metabolism in aged mice, yet it is unknown if treatment is beneficial in the context of obesity or weight loss in adult mice.
- What does this study add?
  ○ Our results suggest that secretome treatment can offset obesity‐related health impairments and enhance the beneficial effects of weight loss. This includes reductions in body and liver adiposity and fibrosis and improvements in muscle cellular qualities and metabolic health.
- How might these results change the direction of research or clinical practice?
  ○ Stem cell‐based secretomes are a promising therapeutic approach to improve obesity related health outcomes with or without dietary intervention.

## 1 Introduction

Obesity is a highly prevalent condition with a global fingerprint resulting in significant health risks [1] including the development of metabolic syndrome [2], type II diabetes [3], and fatty liver diseases [4]. The implementation of modern pharmacotherapies including glucose‐dependent insulinotropic polypeptide (GIP) and glucagon‐like peptide‐1 (GLP‐1) receptor agonists are effective in reducing body weight and improving metabolic outcomes in individuals with overweight and obesity [5, 6] alone or in combination with exercise [7]. Despite their effectiveness, withdrawal from these treatments results in a considerable reversal of weight loss and associated cardiometabolic improvements [8]. Furthermore, there is possible concern regarding the loss of skeletal muscle mass during treatment [9, 10], which may outpace the expected loss from diet or age‐related atrophy [11]. Accordingly, there is rationale for investigating additive or alternative approaches to treat obesity and its related adverse conditions while also preserving or enhancing skeletal muscle mass.

Stem cell‐based therapies have emerged as a powerful tool to treat a wide range conditions and diseases [12, 13] including their ability to elicit secondary actions through the secretion of growth factors, metabolites, chemokines and cytokines, and extracellular vesicles [14, 15]. In fact, stem cell‐derived secretomes have been demonstrated to be beneficial in the context of aging [16, 17] and regenerative medicine [18, 19, 20, 21], while less is known considering the treatment of obesity‐related conditions [22]. Nonetheless, prior work from our lab has shown that 4 weeks of an intramuscular treatment with an embryonic stem cell secretome product improved muscle quality and strength in aged mice and increased whole‐body metabolism and fat loss [16]. Moreover, the secretome product stimulated muscle cell hypertrophy and adipocyte lipolysis through direct and indirect mechanisms in vitro. Therefore, the use of stem cell secretomes may confer beneficial effects in the conditions of obesity and metabolic disease.

In the present study we examined the effects of acute (4 weeks) intramuscular secretome treatment (biweekly) on body composition, metabolic health (glucose handling, fasting insulin), and tissue quality (skeletal muscle, fat pads, liver) in obese mice maintained on a high fat diet as well as obese mice undergoing concurrent weight loss by returning to a standard diet. We hypothesized that the secretome treatment would improve metabolic health and tissue quality as well as enhance weight loss in obese mice maintained on a high fat diet and in obese mice during weight loss.

## 2 Methods

### 2.1 Experimental Design

Male C57BL/6 mice (Jackson Laboratory, Bar Harbor, ME) were used for all experiments beginning at 1 month and were maintained on a standard chow diet (24% protein, 60% carbohydrate, 16% fat kcal/g, 2920X, Envigo Teklad, Madison, WI) or provided with a high fat diet (18.3% protein, 21.4% carbohydrate, 60.3% fat kcal/g, TD.06414, Envigo Teklad) for 16 weeks. High fat diet fed mice were randomized to remain on a high fat diet or return to a chow diet for 4 weeks while receiving biweekly intramuscular injections (50 μL) to the right quadriceps with a vehicle (saline, 0.9% USP) or a stem cell secretome product (40% v/v in saline).

The secretome product was derived in chemically defined media from partially differentiated pluripotent human embryonic stem cells (CSC14, CVCL_B918) free from exogenous proteins including albumin, growth factors, and hormones. Conditioned media was pooled and concentrated by tangential flow filtration and sterile filtered to generate a standardized clinical grade cell free secretome product (IMMUNA, Immunis Inc., Irvine, CA). This product is tested currently under US FDA regulations in a phase II clinical trial (NCT06600581) to improve muscle performance in seniors with obesity and muscle weakness. The secretome contains 1 mg/mL secreted proteins with key components (e.g., fetuin A and follistatin) that are quantitatively monitored across batches and other significantly represented proteins including carrier proteins, growth factors, tissue remodeling and immune factors such as insulin‐like growth factor binding proteins, vascular endothelial growth factors, cadherins, and matrix metallopeptidases, as well as various chemokines and cytokines [18]. The secretome also contains microRNA carrying exosomes which target genes related to the effects described earlier [16]. Pharmacokinetic analysis demonstrates the secretome can be detected in murine plasma for up to 72 h following a single intravenous, intramuscular, or subcutaneous injection.

### 2.2 Body Composition, Performance, and Metabolic Measures

Body weight, tissue composition (Bruker Minispec Mq20 NMR analyzer, Rheinstetten, Germany), and whole‐body grip strength (Grip Strength Meter, Columbus Instruments, Columbus, OH) were recorded weekly. Rotarod performance was recorded at baseline and following treatment, using 0.3 rpm/s acceleration (Rotamex‐5, Columbus Instruments) as the best of three attempts. All animal procedures were conducted in agreement with standards set by the University of Utah Institutional Animal Care and Use Committee. Glucose tolerance testing (0–120 min) occurred following an overnight fast and I.P. injection of glucose (1 g/kg, 10% glucose) and tail bleed assessments. Insulin levels were assessed with an ultra‐sensitive mouse ELISA kit (Cayman Chemical, Ann Arbor, MI) per manufacturer recommendations.

### 2.3 Tissue Collection, Histology, and Immunohistochemistry

A lateral portion of the right quadriceps and whole‐hearts were embedded in optimal cutting temperature compound (OCT, Fisher Scientific, Waltham, MA) and frozen in liquid nitrogen cooled isopentane, stored at −80°C, and sectioned at 10 μm (CM1860, Leica Biosystems, Wetzlar, Germany). Portions of the left lobe of livers as well as fat pads (I‐WAT, E‐WAT, BAT) were placed in 10% formalin for 24–48 h, stored in 70% ethanol at 4°C, paraffin imbedded, and microtome sectioned (5 μm). Paraffin sections of livers and fat pads were dewaxed and stained with H&E. Muscle capillary density was determined by assessing CD31 (550274, BD Biosciences, Franklin Lakes, NJ) and DAPI (1:10,000, D3571, Invitrogen, Waltham, MA) positive cells relative to the number of muscle fibers (WGA, 1:200, W32466, Thermo Fisher Scientific). Tissue fibrosis was assessed by measuring the area of Sirius Red staining (ab246832, Abcam, Cambridge, UK). Brightfield and fluorescent images were obtained at 10‐20X using an Axio scan.z1 (Carl Zeiss, Oberkocken, Germany) or EVOS FL microscope (Thermo Fisher Scientific). Myofiber cross‐sectional area and capillarity density were analyzed using MuscleJ 2.0 and manual analysis [23], fat pad adipocyte size using the Adiposoft plugin [24], and liver adiposity using Fiji by semi‐automated thresholding and particle analysis.

### 2.4 Statistical Analysis

All data are presented as mean ± standard deviation. Data were visually assessed for normality including Q‐Q plots, skewness and kurtosis, and Shapiro‐Wilks tests as warranted. As indicated, data were analyzed using one‐way and two‐way ANOVA including Tukey or Holm‐Bonferroni post hoc comparisons where relevant. If any data were missing, a mixed effects alternative was employed. *T*‐tests were used to compare two groups at singular time points. Significance was set as *p* < 0.05 with * = *p* < 0.05, ** < 0.01, and *** < 0.001 throughout. GraphPad Prism (v10.4.2) and Excel (2024) were used for all analysis and graphical composition. One chow liver and one HFD E‐WAT sample were inadequate for histological analysis.

## 3 Results

### 3.1 Secretome Treatment Improves Body Composition During Obesity and Weight Loss Conditions

**FIGURE 1:**
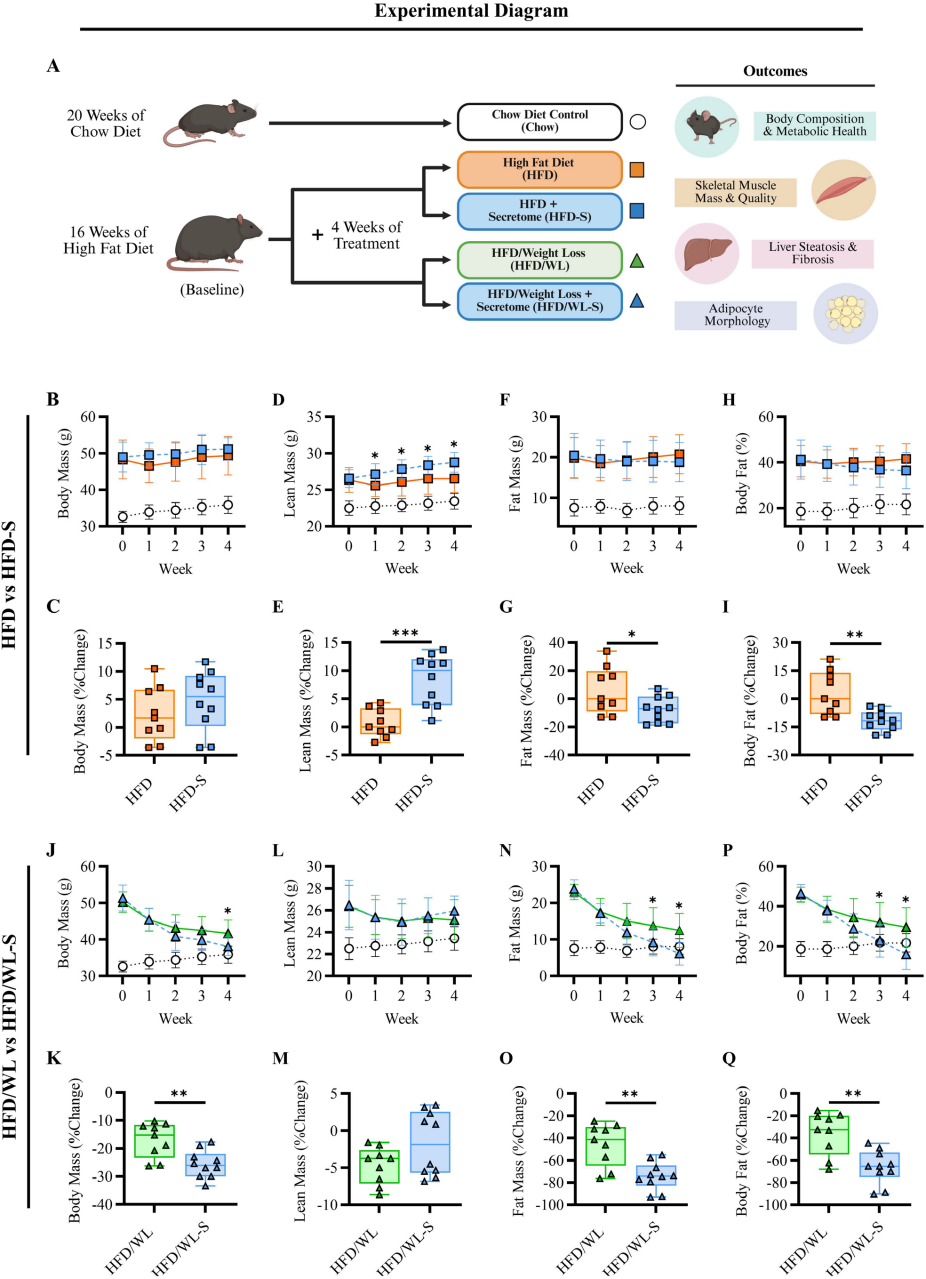
Weekly body composition. (A) Experimental diagram. (B–I) Weekly and percent change at 4 weeks from baseline in body composition; body (g), lean (g), and fat mass (g), as well as body fat %, for high fat diet (HFD, n:9) and high fat diet secretome treated (HFD‐S, n:9) compared to chow control (Chow, n:10) mice. (J–Q) Weekly and percent change at 4 weeks from baseline in body composition for weight loss (HFD/WL, n:9) and weight loss secretome treated (HFD/WL‐S, n:10) compared to Chow mice. Chow mice shown as white circles, HFD mice as orange squares, HFD‐S mice as blue squares, HFD/WL mice as green triangles, and HFD/WL‐S mice as blue triangles. Panels B, D, F, H, J, L, N, and P analyzed via two‐way ANOVA and panels C, E, G, I, K, M, O, and Q with *T*‐tests. * = significant difference between groups, * < 0.05, ** < 0.01, *** < 0.001.

To induce obesity, mice completed 16 weeks of ad libitum high fat diet feeding resulting in significant gain of body weight, fat and lean mass, and body fat % (Figure S1A–D). A subset of obese mice were maintained on a high fat diet and randomized to receive either saline treatment (HFD) or secretome treatment for 4 weeks (HFD‐S) (Figure 1A). Additional groups of obese mice underwent weight loss by returning to a standard chow diet over a 4‐week period during which they received either saline (HFD/WL) or secretome treatment (HFD/WL‐S) (Figure 1A).

During sustained high fat diet feeding, HFD and HFD‐S mice had similar weekly body mass and were heavier than chow mice (Figure 1B,C). Conversely, HFD‐S mice had increased lean mass compared to HFD mice across 4 weeks of treatment (Figure 1D) as well as greater relative (% change) lean mass increases at 4 weeks compared to baseline (Figure 1E). While weekly fat mass did not robustly differ between HFD and HFD‐S groups (Figure 1F), HFD‐S mice had greater relative decreases in fat mass at week 4 compared to baseline (Figure 1G). Similarly, HFD and HFD‐S mice had similar body fat % at all time points (Figure 1H) while HFD‐S mice had greater decreases in relative body fat % by week 4 compared to baseline (Figure 1I).

During dietary weight loss following high fat diet feeding both HFD/WL and HFD/WL‐S mice reduced body weight but the relative change was greater for HFD/WL‐S vs. HFD/WL mice at week 4 (Figure 1J,K). Additionally, body weight for HFD/WL‐S (*p* = 0.20) but not HFD/WL (*p* < 0.01) mice was similar to chow levels. Weekly lean mass was not different between groups (Figure 1L,M). While both HFD/WL and HFD/WL‐S mice showed decreased fat mass during treatment, this was more pronounced in HFD/WL‐S mice who had greater absolute and relative fat mass reductions compared to HFD/WL mice (Figure 1N,O). In fact, at week 4, HFD/WL‐S mice had similar fat mass to chow mice (*p* = 0.28), but HFD/WL fat mass was increased (*p* < 0.01) compared to chow levels. Likewise, weekly reductions in body fat % were greater for HFD/WL‐S compared to HFD/WL mice at weeks 3 and 4 and considering relative change from baseline (Figure 1P,Q), with HFD/WL‐S (*p* = 0.06) but not HFD/WL (*p* = 0.04) displaying similar body fat % compared to chow mice at week 4.

Together, these results demonstrate that secretome treatment improves body composition in obese mice on a high fat diet as well as during weight loss. While remaining on a high fat diet, secretome treatment increased lean mass but was less effective in reducing fat mass. Conversely, during weight loss, secretome treatment greatly improved body composition by accelerating the loss of fat mass.

### 3.2 Secretome Treatment Improves Glucose Handling and Fasting Insulin Following Weight Loss

**FIGURE 2:**
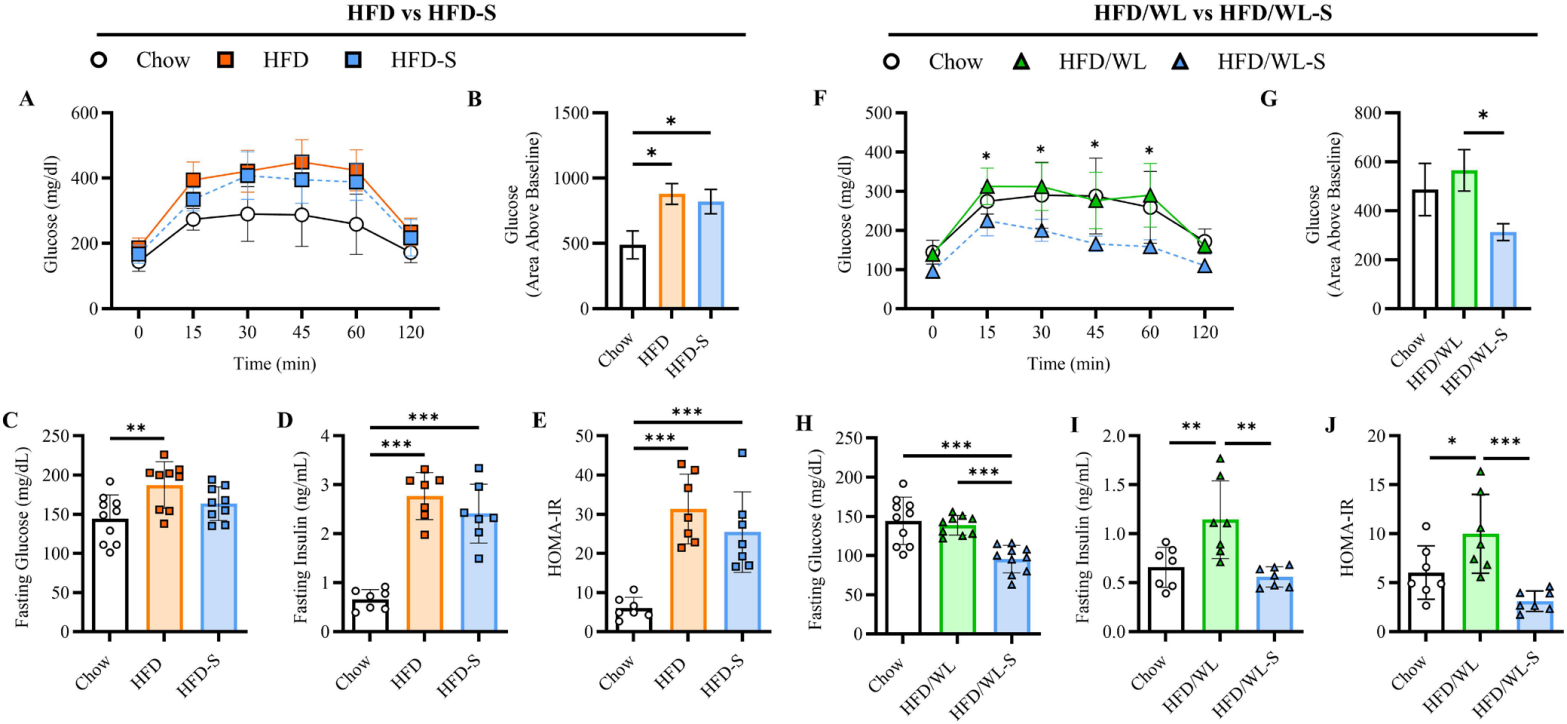
Glucose handling and insulin levels. (A, B) I.P. glucose handling (mg/dL) over 120 min and glucose area above baseline for high fat diet (HFD, n:9) and high fat diet secretome treated (HFD‐S, n:9) compared to chow control (Chow, n:10) mice. (C–E) Fasting glucose (mg/dL), insulin (ng/mL, n:7), and calculated HOMA‐IR (n:7) for HFD, HFD‐S, and Chow mice. (F, G) 120 min glucose handling and area above baseline for weight loss (HFD/WL, n:9) and weight loss secretome treated (HFD/WL‐S, n:10) mice compared to Chow mice. (H–J) Fasting glucose, insulin (n:7), and calculated HOMA‐IR (n:7) for HFD/WL, HFD/WL‐S, and Chow mice. Chow mice shown as white circles, HFD mice as orange squares, HFD‐S mice as blue squares, HFD/WL mice as green triangles, and HFD/WL‐S mice as blue triangles. All data collected at the 4‐week time point. Panels A and B analyzed via two‐way ANOVA and panels B‐E and G‐P via one‐way ANOVA. * = significant difference between groups, * < 0.05, ** < 0.01, *** < 0.001.

As secretome treatment enhanced lean mass or accelerated fat mass loss in obese mice with or without dietary intervention, we also examined changes in glucose homeostasis and fasting insulin concentrations. In the first experiment, HFD and HFD‐S mice had impaired glucose tolerance compared to chow mice at 4 weeks (Figure 2A,B) that were not different from one another at baseline or following treatment (Figure S2A,B) although there were modest reductions in fasting glucose levels for HFD‐S animals (Figure 2C). Likewise, fasting insulin and HOMA‐IR were not different between HFD‐S and the HFD group and were significantly elevated relative to chow control mice (*p* < 0.05) (Figure 2D,E).

In mice switched to standard chow, the glucose tolerance of HFD/WL and HFD/WL‐S mice was similar at baseline and improved following treatment while glucose area above baseline was only improved at 4 weeks for HFD/WL‐S mice (Figure S2B,C). Remarkably, while the 4‐week glucose tolerance of HFD/WL mice was similar to that of chow mice, HFD/WL‐S experienced further improvements in glucose handing compared to both chow and HFD/WL mice (Figure 2F,G). Similarly, fasting glucose levels were reduced in HFD/WL‐S mice compared to HFD/WL and chow mice (Figure 2H). Moreover, fasting insulin and HOMA‐IR were significantly lower in HFD/WL‐S compared to HFD/WL mice and were indistinguishable from chow levels (Figure 2I,J).

Therefore, while these data demonstrate secretome treatment had little effect on glucose handling and fasting insulin levels in obese mice that remained on a high fat diet, there were substantial improvements to all metabolic parameters when secretome treatment was combined with weight loss.

### 3.3 Secretome Mediates Changes in Tissue Compartments During Obesity and Weight Loss

To elucidate the secretome‐mediated changes in body composition and glucose handling, we evaluated tissue weights of dissected skeletal muscle (quadriceps, gastrocnemius, soleus), fat pads (I‐WAT, E‐WAT, BAT), and organs (liver, heart, spleen) at the end of 4 weeks of treatment. Data were corrected to body weight (BW) and are also presented as absolute values in Figure S3.

**FIGURE 3:**
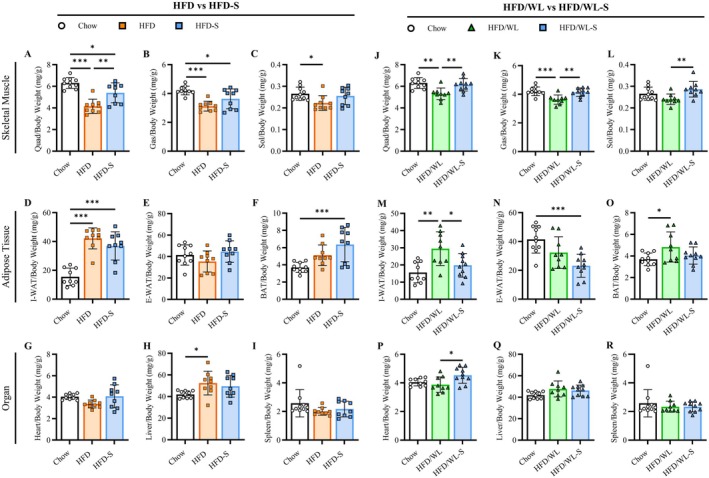
Relative tissue masses. (A–C) Average quadriceps, gastrocnemius, and soleus muscle mass relative to total body weight (mg) for high fat diet (HFD, n:9) and high fat diet secretome treated (HFD‐S, n:9) compared to chow control (Chow, n:10) mice. (D–F) Average inguinal (I‐WAT), epididymal (E‐WAT), and BAT fat pad mass relative to total body weight for HFD, HFD‐S, and Chow mice. (G–I) Average quadriceps, gastrocnemius, and soleus muscle mass relative to total body weight for weight loss (HFD/WL, n:9) and weight loss secretome treated (HFD/WL‐S, n:10) compared to Chow mice. (J–L) Average I‐WAT, E‐WAT, and BAT fat pad mass relative to total body weight for HFD/WL, HFD/WL‐S, and Chow mice. Chow mice shown as white circles, HFD mice as orange squares, HFD‐S mice as blue squares, HFD/WL mice as green triangles, and HFD/WL‐S mice as blue triangles. All data collected at the 4‐week time point. Analyzed using one‐way ANOVA. * = significant difference between groups, * < 0.05, ** < 0.01, *** < 0.001.

In mice maintained on a high fat diet, both HFD and HFD‐S mice had reduced quadriceps muscle/BW ratios compared to chow controls, yet the loss in skeletal muscle weight was remarkably attenuated in HFD‐S mice compared to HFD mice (Figure 3A). Changes in gastrocnemius and soleus were not different between HFD and HFD‐S groups (Figure 3B,C). Compared to chow mice, HFD and HFD‐S animals had increased I‐WAT/BW but not E‐WAT/BW ratios, while only HFD‐S mice had increased BAT/BW ratios (Figure 3D–F). There were no differences in relative heart, liver, or spleen weight between HFD and HFD‐S mice, yet HFD mice had increased liver ratios compared to chow mice (Figure 3G–I).

In mice switched to standard chow HFD/WL mice had reduced muscle/BW ratios compared to chow mice, while secretome treated HFD/WL‐S mice did not (Figure 3J–L). As HFD/WL‐S mice did not display differences in absolute muscle weights this appears primarily driven by reductions in body or fat mass (Figure S3J–L). HFD/WL‐S mice also had lower I‐WAT/BW and E‐WAT/BW ratios than HFD/WL mice but similar BAT/BW ratios compared to chow mice (Figure 3M–O). On the other hand, heart/BW ratios were increased in HFD/WL‐S mice compared to HFD but not chow mice, while liver and spleen ratios did not differ between HFD/WL, HFD/WL‐S, and chow groups, respectively (Figure 3P–R).

Cumulatively, secretome treatment mediated beneficial changes to relative tissue weights in obese mice during high fat diet feeding and weight loss. HFD‐S mice displayed increased muscle weights which were less robust in HFD/WL‐S mice. Alternatively, adipose/BW levels and absolute masses were restored to those of chow control mice when secretome treatment was administered concurrent with dietary weight loss. Individual muscle weights from injected and contralateral limbs are available in Table S1 and corroborate the combined muscular effects.

### 3.4 Secretome Treatment Improves Muscle Cellular Size and Quality

We have previously demonstrated that intramuscular secretome treatment improved myofiber size and cellular remodeling in aged mice [16] and during muscle regrowth [18]. Building on these findings, we explored the effect of secretome treatment on muscle myofiber size, capillarization, and collagen content as well as physical performance outcomes in the current investigation.

**FIGURE 4:**
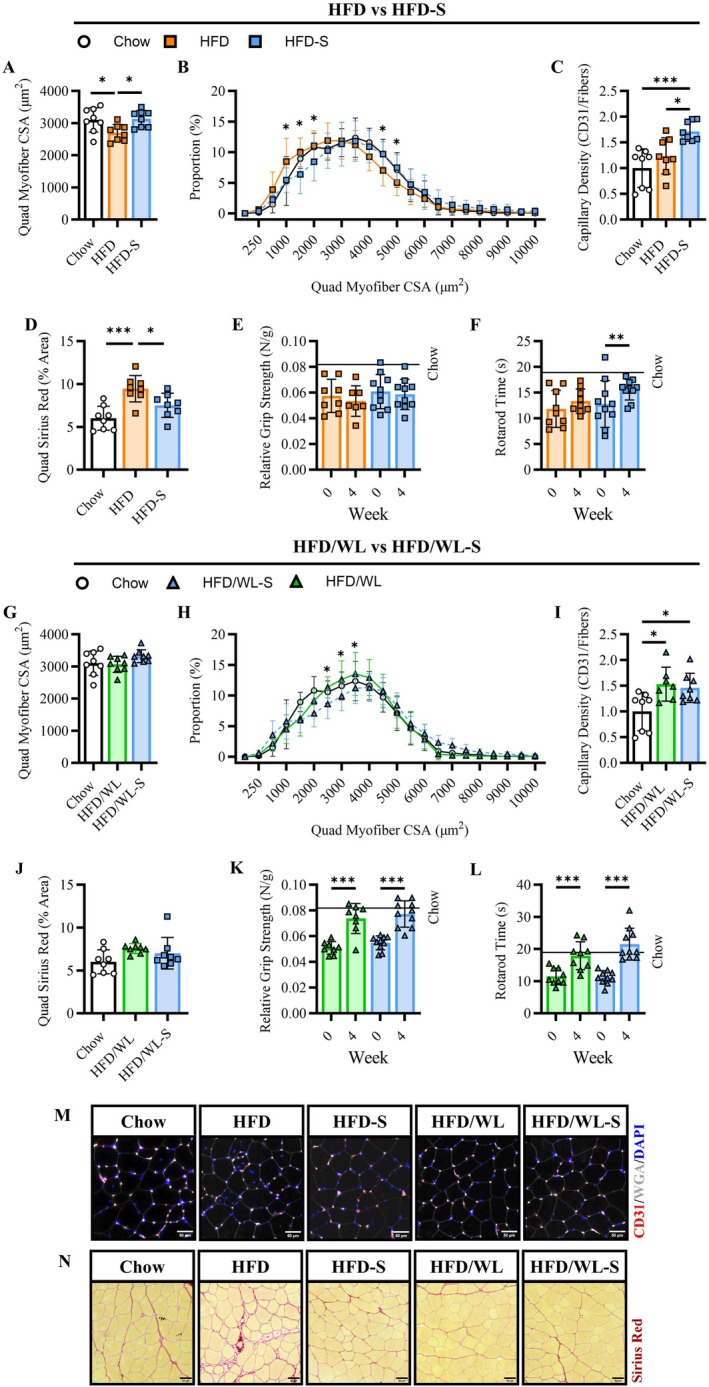
Muscle cellular size and qualities. (A, B) Right quadriceps average myofiber cross‐sectional area (CSA, μm^2^) and size distribution (%) for high fat diet (HFD, n:8) and high fat diet secretome treated (HFD‐S, n:8) compared to chow control (Chow, n:8) mice. (C, D) Quadriceps capillary density (CD31 + DAPI+ cells/total fibers) and Sirius Red area (%) for HFD, HFD‐S, and Chow mice. (E, F) Average grip strength relative to total body weight and rotarod time. (G, H) Right quadriceps average myofiber CSA and size distribution for weight loss (HFD/WL, n:8) and weight loss secretome treated (HFD/WL‐S, n:8) mice compared to Chow mice. (I, J) Quadriceps capillary density (CD31 + DAPI+ cells/total fibers) and % Sirius Red area for HFD, HFD‐S, and Chow mice. (K, L) Average grip strength relative to total body weight and rotarod time. (M, N) Representative images for capillary density (WGA: white, CD31: red, DAPI: blue) and Sirius Red staining (scale: 50 μm). Chow mice shown as white circles, HFD mice as orange squares, HFD‐S mice as blue squares, HFD/WL mice as green triangles, and HFD/WL‐S mice as blue triangles. All data collected at the 4‐week time point. Panels A, C, D, G, I, and J analyzed using one‐way ANOVA and panels B, E, F, H, K, and L using two‐way ANOVA. * = significant difference between groups, * < 0.05, ** < 0.01, *** < 0.001.

During continued high fat diet feeding, average quadriceps fiber size was greater in HFD‐S compared to HFD mice which had reduced fiber size compared to chow mice (Figure 4A). This was further supported by a rightward shift in myofiber size distribution in HFD‐S compared to HFD mice (Figure 4B). Similarly, compared to chow controls, HFD mice had a greater proportion of small fibers (500–1000 μm^2^) and fewer large fibers (4000–4500 μm^2^) while HFD‐S mice had fewer small fibers (1000–2000 μm^2^) but greater large fibers (4500–5000 μm^2^). HFD‐S mice also displayed markedly increased quadriceps capillary density compared to both chow and HFD mice (Figure 4C,M). Interestingly, there was a substantial mitigation of quadriceps fibrosis as indicated by lower Sirius Red content in HFD‐S compared to HFD mice, with secretome treatment reducing fibrosis similar to the levels of chow mice (Figure 4D,N). These alterations to muscle quality did not correspond to differences in absolute grip strength at 4 weeks (2.82 ± 0.61 vs. 2.98 ± 0.55 N, *p* = 0.55) or relative (N/BW) change from baseline (Figure 4E) comparing HFD and HFD‐S mice, respectively. In contrast, secretome treatment improved rotarod performance which was similar to chow levels at week 4 in HFD‐S but not HFD mice (Figure 4F).

In mice switched to a standard chow diet, average quadriceps fiber size was not significantly different between any group (Figure 4G). Alternatively, there was a modest rightward fiber distribution shift in HFD/WL‐S compared to HFD/WL mice which had a greater proportion of small fibers (1500 μm^2^) compared to chow controls while HFD‐S mice had fewer small fibers (1500–2000 μm^2^) (Figure 4H). Neither capillary density nor collagen content differed between HFD/WL and HFD/WL‐S groups (Figure 4I,J,M,N). Relative grip strength and rotarod performance increased during weight loss (Figure 4K,L) similar to HFD/WL and HFD/WL‐S mice and absolute grip strength was not different at week 4 (3.14 ± 0.49 vs. 2.88 ± 0.52 N, *p* = 0.27), respectively.

Together, these data demonstrate improvements in muscle fiber size and collagen content (muscle quality) in obese high fat diet fed mice with modest translation to rotarod performance following secretome treatment. In addition, when combined with dietary weight loss, secretome treatment modestly improved proportional muscle fiber size in obese mice.

### 3.5 Liver Fibrosis and Tissue Adiposity Are Ameliorated in Secretome Treated Mice

**FIGURE 5:**
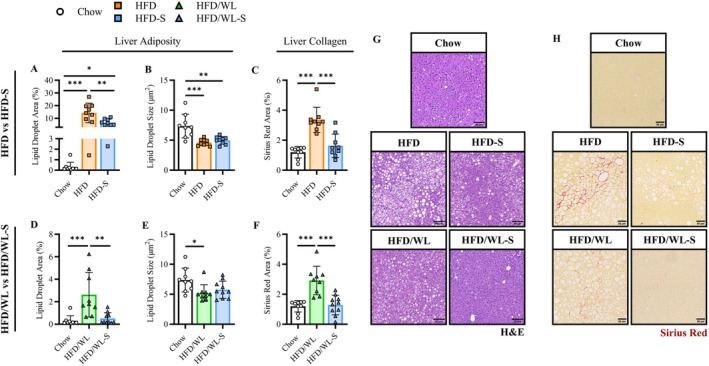
Liver steatosis and fibrosis. (A–C) Average liver lipid droplet area (%), lipid droplet size (μm^2^), and Sirius Red area (%) for high fat diet (HFD, n:9) and high fat diet secretome treated (HFD‐S, n:9) compared to chow control (Chow, n:9) mice. (D–F) Average liver lipid droplet area, lipid droplet size, and Sirius Red area for weight loss (HFD/WL, n:9) and weight loss secretome treated (HFD/WL‐S, n:10) mice compared to Chow mice. (G, H) Representative images for liver H&E and Sirius Red staining (scale: 50 μm). Chow mice shown as white circles, HFD mice as orange squares, HFD‐S mice as blue squares, HFD/WL mice as green triangles, and HFD/WL‐S mice as blue triangles. All data collected at the 4‐week time point. Analyzed using one‐way ANOVA. * = significant difference between groups, * < 0.05, ** < 0.01, *** < 0.001.

Considering the reductions in adipose mass and muscle fibrosis with secretome treatment, we next performed histological analysis to evaluate the effects of secretome treatment on liver steatosis, liver fibrosis, and adipocyte morphology in fat pads. HFD mice had substantially greater liver lipid content compared to chow mice including both micro‐ and macrovesicular accumulation which was globally attenuated in HFD‐S mice (Figures 5A,B,G and S4A). Liver fibrosis, as indicated by Sirius Red staining, was elevated in HFD mice compared to HFD‐S and chow mice, while liver fibrosis in HFD‐S mice was similar to chow levels (Figure 5C,H). Of note, collagen dense areas were localized within adipocyte rich liver regions. When switched to a standard chow diet, liver adiposity in HFD/WL‐S mice was similar to chow controls while HFD/WL mice increased adiposity compared to both chow and HFD‐S mice (Figure 5D,G). In parallel, lipid droplet size and density of HFD/WL‐S but not HFD/WL livers had returned to chow levels (Figures 5E and S4B). Furthermore, secretome treatment substantially reduced liver fibrosis in HFD/WL‐S mice (vs HFD/WL) and was similar to chow mice (Figure 5F,H).

**FIGURE 6:**
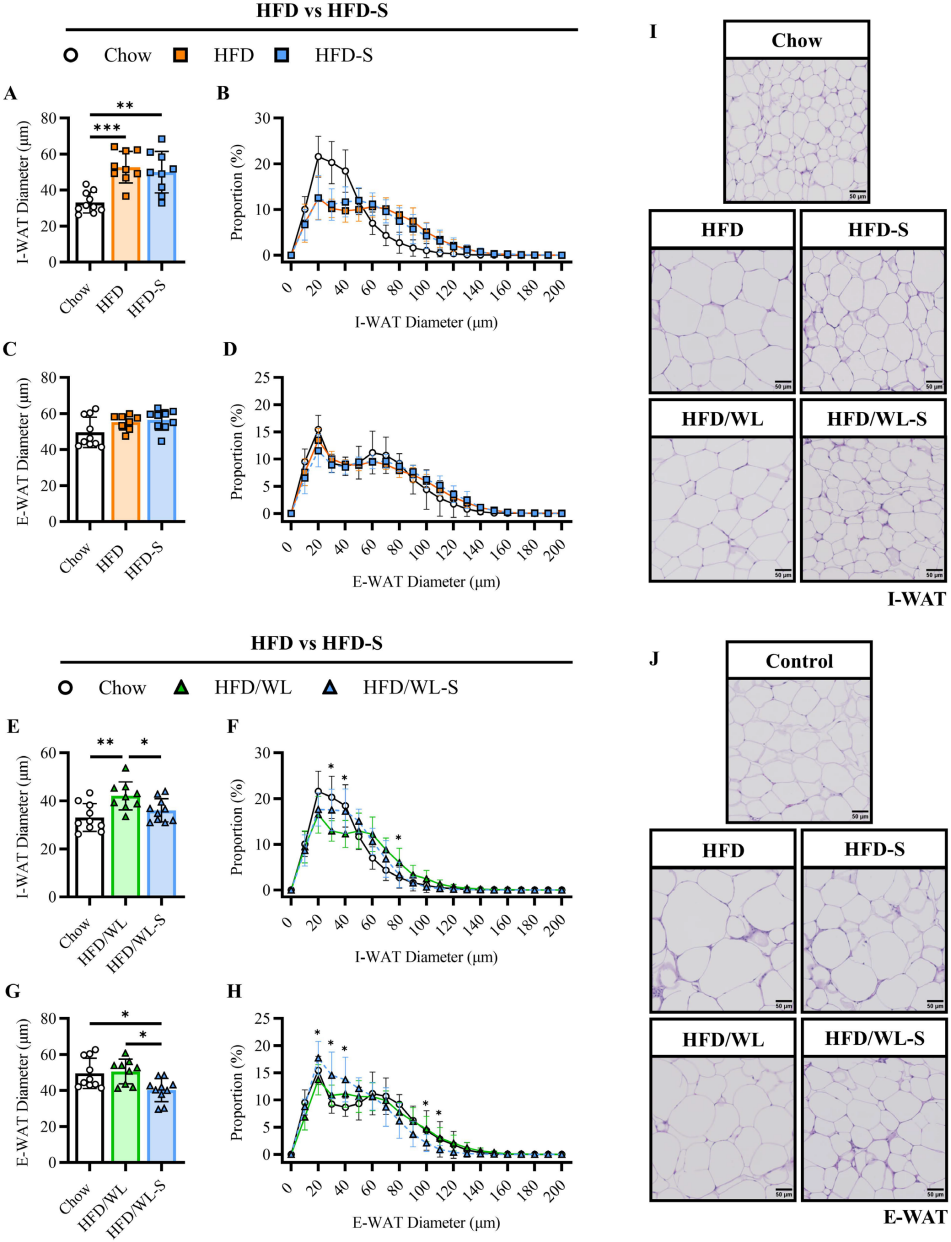
Body fat characteristics. (A–D) Average adipocyte diameter (μm) and size distribution (%) for inguinal (I‐WAT) and epididymal (E‐WAT) fat pads for high fat diet (HFD, n:7–8) and high fat diet secretome treated (HFD‐S, n:9) compared to chow control (Chow, n:9) mice. (E–H) Average adipocyte diameter (μm) and size distribution for I‐WAT and epididymal E‐WAT fat pads for high fat diet weight loss (HFD/WL, n:9) and high fat diet weight loss secretome treated (HFD/WL‐S, n:10) compared to Chow mice. (I, J) Representative images for I‐WAT and E‐WAT H&E staining (scale: 50 μm). Chow mice shown as white circles, HFD mice as orange squares, HFD‐S mice as blue squares, HFD/WL mice as green triangles, and HFD/WL‐S mice as blue triangles. All data collected at the 4‐week time point. Analyzed using one‐way ANOVA. * = significant difference between groups, * < 0.05, ** < 0.01, *** < 0.001.

While maintained on a high fat diet, HFD and HFD‐S mice had relatively similar alterations in I‐WAT and E‐WAT adipocyte size including rightward distribution shifts compared to chow controls (Figure 6A–D,I,J). In mice switched to a standard chow diet, HFD/WL‐S I‐WAT adipocytes were smaller compared to HFD/WL mice while E‐WAT adipocytes were smaller compared to both HFD/WL and chow mice (Figure 6E–H,J). We additionally investigated changes in BAT adipocyte morphology in a subset of mice and found no differences between groups (Figure S4C–G). As we previously observed increases in heart mass with secretome treatment, we also examined myocardial morphology in a subset of mice. Notably, there were no differences in left ventricular wall thickness or cardiomyocyte diameter comparing HFD and HFD‐S or HFD/WL and HFD/WL‐S groups (Figure S3H–L).

Thus, these results show that secretome treatment reduces liver steatosis and improves tissue fibrosis in obese mice remaining on a high fat diet. Moreover, liver adiposity and quality are further improved by secretome treatment during weight loss along with pronounced reductions in fat pad adipocyte sizes.

## 4 Discussion

In the present investigation we demonstrate that 4 weeks of intramuscular secretome treatment during high fat feeding reduced liver steatosis and fibrosis while improving skeletal muscle fiber size and collagen content. Moreover, secretome treatment enhanced the effects of dietary induced weight loss on whole‐body adiposity, liver steatosis, and fibrosis, as well as fasting insulin levels and glucose handling, without reducing skeletal muscle mass. Together, these findings suggest that short term treatment with a secretome product improves basal health status in obesity and amplifies the positive effects of dietary weight loss.

A major finding of the current study includes secretome treatment mediated reductions in liver steatosis in mice maintained on a high fat diet and a full return to chow control levels during dietary weight loss. Notably, there was both micro‐ and macrovesicular lipid droplet accumulation in high fat diet fed mice, which is indicative of significant liver dysfunction, metabolic stress, and disease progression [25]. Liver fibrosis is a major consequence of obesity and steatosis [26] but was absent in both secretome treated groups despite elevations in their untreated counterparts. These findings are particularly intriguing considering steatosis and fibrosis underscore metabolic dysfunction‐associated fatty liver disease (MAFLD) [27], which has substantial global prevalence [4] and burden [28]. Furthermore, in patients with fatty liver disease, fibrosis is a strong independent predictor of lifelong health outcomes and mortality [29, 30]. There is currently a single FDA approved pharmaceutical intervention for advanced forms of fatty liver disease [31], while GLP‐1 receptor agonists show encouraging results [32]. Therefore, secretome treatment shows promise to improve liver health alone or in combination with weight loss in the context of obesity.

We previously demonstrated that a secretome product reduced whole‐body fat mass in aged mice, seeding the premise that this novel treatment may offer benefits in overweight and obesity conditions [16]. Our current results now demonstrate that secretome treatment combined with dietary weight loss provided robust reductions in whole‐body adiposity. These changes included smaller fat mass depots and adipocyte size compared to the untreated weight loss group and, in some parameters (E‐WAT), further reductions compared to chow controls. While our previous work shows that the secretome product can stimulate lipolysis directly in vitro, it can also indirectly affect secondary tissues such as adipocytes through the release of myokines from skeletal muscle cells [16]. Therefore, we speculate that secretome treatment improves adiposity not only through muscle mediated actions but also directly reaches fatty tissues through circulation. A systemic action following secretome treatment would also help explain the observed reductions in liver steatosis suggesting that the liver could be a primary beneficiary in the present investigation due to its proximal role in systemic circulation. This possibility highlights the potential of secretome treatment in obesity‐related metabolic disease states such as those with prominent liver pathologies (e.g., MASH).

Another major finding in this study includes robust improvements in glucose tolerance and fasting insulin during weight loss combined with secretome treatment, possibly due to the adiposity reductions across multiple tissues (adipose, liver). The lack of treatment effect on metabolic parameters in the HFD group was surprising given the large reductions in liver steatosis, which independently predicts metabolic dysfunction [33]. Alternatively, research suggests reductions in visceral but not inguinal fat improve insulin sensitivity and glucose tolerance in obese or aged rats [34, 35]. Therefore, the small changes in fat mass in secretome‐treated HFD mice could partially explain the lack of response on metabolic outcomes. Conversely, secretome treatment during concurrent high fat feeding may require a longer intervention period to elicit beneficial metabolic adaptations. Nonetheless, the positive improvements to glucose handling and fasting insulin following secretome treatment during dietary weight loss show promising therapeutic potential for metabolically compromised states.

Finally, in agreement with our previous research in aged mice [16, 18], secretome treatment increased muscle quality including greater myofiber size and capillary density and reduced fibrosis in HFD mice. Additionally, despite substantial reductions in body and fat mass following dietary weight loss with secretome treatment, there were no declines in skeletal muscle mass, which may be relevant to complement emerging pharmacological weight loss interventions including GIP and GLP‐1 receptor agonist. While these treatments are undeniably effective strategies to reduce body weight and enhance metabolism for individuals affected by obesity [5, 6], there are concerns regarding the loss of skeletal muscle mass and function [9, 10, 11, 36] and function [37] during pharmacological intervention which may exceed losses from caloric restriction [38]. Growing evidence also suggests that GLP‐1 therapies can be coupled with hypertrophy inducing agents to further enhance fat loss while preserving or increasing lean mass [39, 40]. Accordingly, our current findings provide rationale for investigating the additive effects of secretome treatment in combination with obesity pharmacotherapies.

In summary, our findings suggest that biweekly intramuscular treatment with a stem cell‐derived secretome product improves liver and muscle quality during high fat diet feeding and further potentiates fat loss and improves metabolic outcomes during diet‐induced weight loss in obese mice. Therefore, secretome treatment may provide benefit to individuals with obesity with impaired liver or muscle quality. Furthermore, secretome treatment appears to heighten the beneficial effects of dietary weight loss without inducing muscular declines. Future studies are warranted to explore the beneficial effects of secretome treatment in combination with other weight loss interventions including exercise or pharmaceutical therapies to improve tissue quality and metabolic outcomes.

## Author Contributions

This project was conceptualized by M.J.D. and designed by M.J.D., Z.J.F., S.A.G., H.S.K., G.N., N.C.B., and T.E.L. Z.J.F., A.S.K., P.‐E.B., C.M.S., R.J.C., E.M.Y., and S.A.G. performed data collection. The manuscript was written by Z.J.F. and M.J.D. and edited by all authors.

## Conflicts of Interest

S.A.G., H.S.K., G.N., N.C.B., T.E.L. are employed by Immunis. M.J.D. serves on the Immunis scientific advisory board. The other authors declared no conflicts of interest.

## Supporting information


**Data S1:** oby70068‐sup‐0001‐Supinfo.pdf.

## Data Availability

The data that support the findings of this study are available from the corresponding author upon reasonable request.
