# Supplementary material for: Stem Cell Secretome Treatment Reduces Adiposity and Improves Glucose Handling During Obesity and Weight Loss in Mice
Source: Obesity (Silver Spring). 2025 Oct 27;34(2):382–93. doi: 10.1002/oby.70068 (PMC12850608; doi:10.1002/oby.70068)
Supplement: Supplementary file 1 — Data S1: oby70068‐sup‐0001‐Supinfo.pdf. [file OBY-34-382-s001.pdf]

Stem cell secretome treatment reduces adiposity and improves glucose handling during obesity and weight loss in mice

Zachary J. Fennel[1,2,3], Anu S. Kurian[1], Paul-Emile Bourrant[1,4], Chad M. Skiles[1,2,3], Robert J. Castro[1,4], Elena M. Yee[1,4], Scott A. Greilach[5], Hans S. Keirstead[5], Gabriel Nistor[5], Nicole C. Berchtold[5], Thomas E. Lane[5,6], Micah J. Drummond[1,2,3,4].

1. Diabetes & Metabolism Research Center, University of Utah, Salt Lake City, UT.
2. Department of Physical Therapy and Athletic Training, University of Utah, Salt Lake City, UT.
3. Molecular Medicine Program, University of Utah, Salt Lake City, UT.
4. Department of Nutrition & Integrative Physiology, University of Utah, Salt Lake City, UT.
5. Immunis, Inc., Irvine California, USA.
6. Department of Neurobiology and Behavior, University of California, Irvine, California, USA.

Micah J. Drummond, University of Utah, 801-585-1310, micah.drummond@hsc.utah.edu

Supplementary Figures

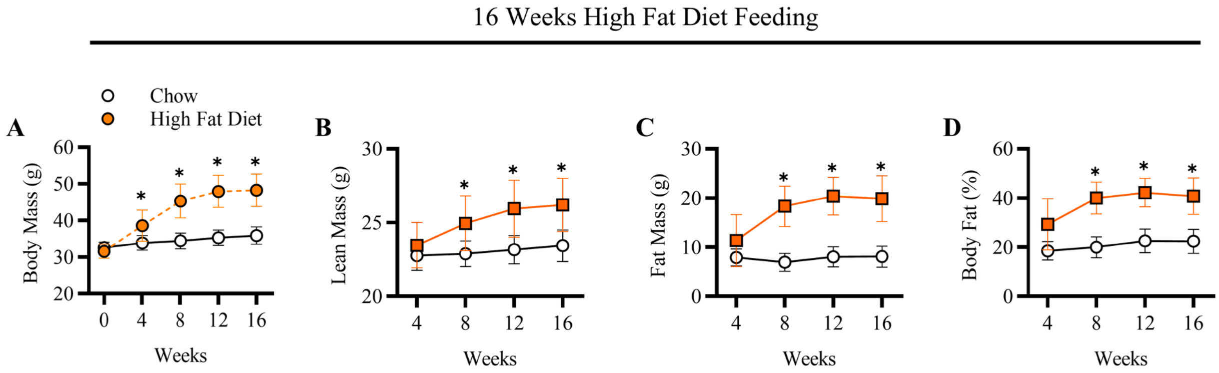

Figure S1. 16 Weeks High Fat Diet Feeding. A-E: Body weight (g), fat mass (g), lean mass (g), and body fat (%) for chow control and high fat diet mice across 16 weeks. Analyzed using two-way ANOVAs. * = significant difference between groups, * <0.05, ** <0.01, *** <0.001

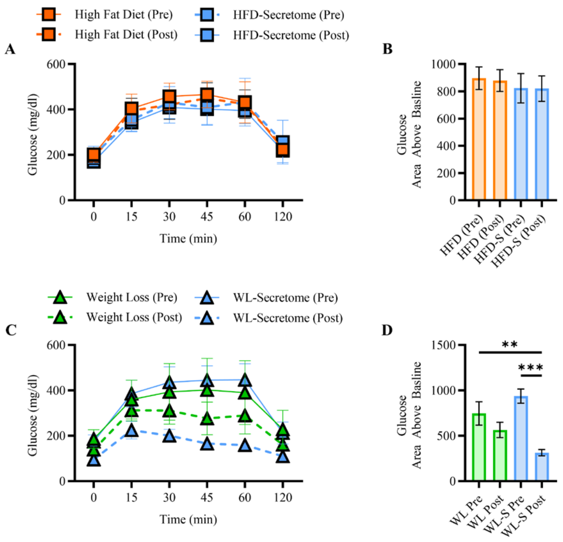

Figure S2. Baseline and 4-Week Glucose Handling. **A** and **B**: I.P. glucose handling (mg/dL) over 120 min and glucose area above baseline for high fat diet (HFD, n:9) and high fat diet secretome treated (HFD-S, n:9) mice at baseline and following 4-weeks of secretome treatment. **C** and **D**: 120 min glucose handling and area above baseline for weight loss (HFD/WL, n:9), and weight loss secretome treated (HFD/WL-S, n:10) mice at baseline and following 4-weeks of secretome treatment. HFD mice shown as orange squares, HFD-S mice as blue squares, HFD/WL mice as green triangles, and HFD/WL-S mice as blue triangles. Solid line indicates baseline, dashed line indicates 4-weeks. Analyzed via two-way ANOVA. * = significant difference between groups, * <0.05, ** <0.01, *** <0.001.

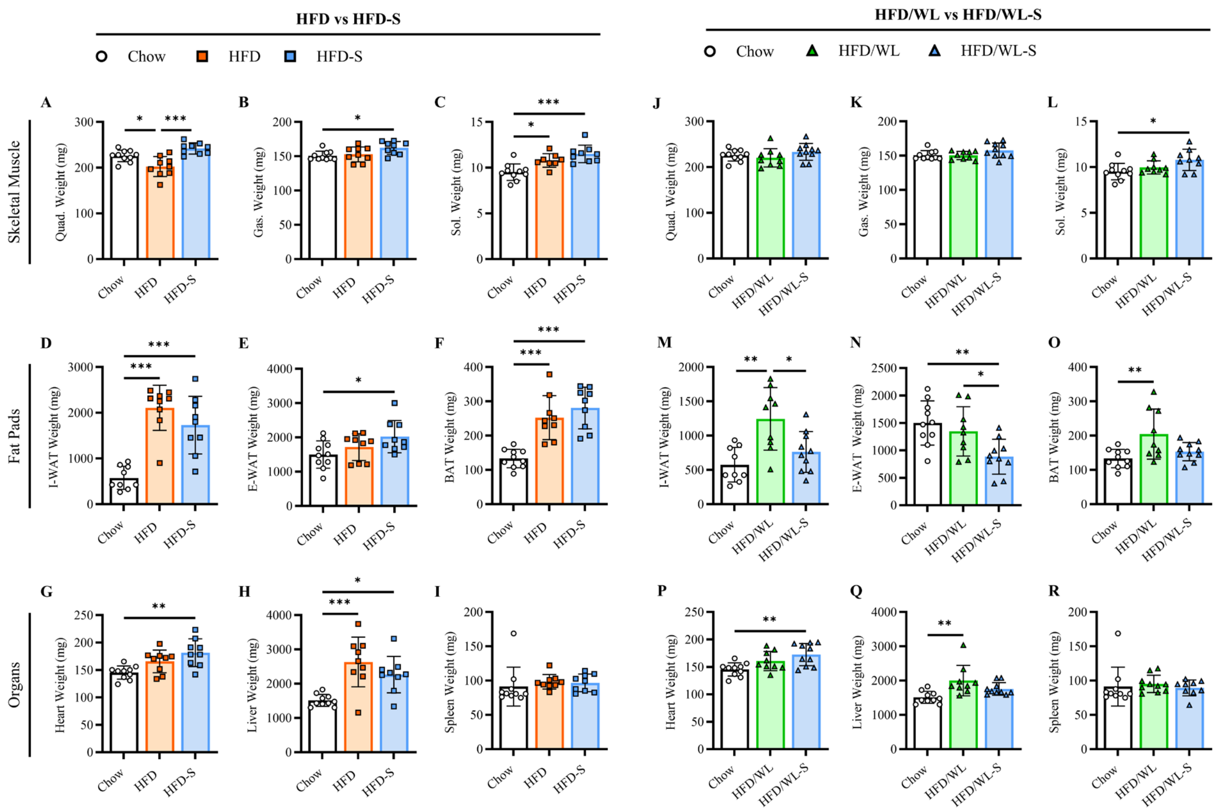

Figure S3. Absolute Tissue Masses. A-C: Average quadriceps, gastrocnemius, and soleus muscle mass (mg) for high fat diet (HFD, n:9) and high fat diet secretome treated (HFD-S, n:9) compared to chow control (Chow, n:10) mice. **D-F**: Average inguinal (I-WAT), epididymal (E-WAT), and brown adipose tissue (BAT) fat pad mass (mg) for HFD, HFD-S, and Chow mice. **G-I**: Average heart, liver, and spleen mass (mg) for HFD, HFD-S, and Chow mice. **J-L**: Average quadriceps, gastrocnemius, and soleus muscle mass for weight loss (HFD/WL, n:9), and weight loss secretome treated (HFD/WL-S, n:10) compared to Chow mice. **M-O**: Average I-WAT, E-WAT, and BAT fat pad mass for HFD/WL, HFD/WL-S, and Chow mice. **P-R**: Average heart, liver, and spleen mass for HFD/WL, HFD/WL-S, and Chow mice. All data collect at the 4-week timepoint. Analyzed using one-way ANOVAs. * = significant difference between groups, * <0.05, ** <0.01, *** <0.001.

Table S1. Individual Muscle Weights.

Muscle	Group	Right	Left	Average
Quadriceps	**Chow**	226.6 ± 13.2	225.2 ± 17.2	225.9 ± 12.5
	**HFD**	199.4 ± 30.6	205.5 ± 37.2	202.4 ± 21.8#
	**HFD-S**	239.9 ± 17.7*	244.3 ± 16.6*	242.2 ± 12.5*
	**HFD/WL**	218.9 ± 19.9	221.8 ± 23.8	220.4 ± 20.2
	**HFD/WL-S**	232.4 ± 13.7	234.0 ± 28.3	233.4 ± 18.3
Gastrocnemius	**Chow**	151.0 ± 8.4	150.5 ± 9.1	150.8 ± 6.6
	**HFD**	151.0 ± 13.9	154.7 ± 12.3	154.3 ± 10.9
	**HFD-S**	160.3 ± 9.9	164.4 ± 11.2#	162.4 ± 9.3#
	**HFD/WL**	147.8 ± 4.9	153.0 ± 8.9	150.4 ± 6.0
	**HFD/WL-S**	156.6 ± 8.7	159.1 ± 14.8	157.9 ± 10.8
Soleus	**Chow**	9.6 ± 1.2	9.4 ± 0.6	9.5 ± 0.9
	**HFD**	10.6 ± 0.5	10.7 ± 0.7	10.7 ± 0.6#
	**HFD-S**	11.6 ± 0.7#	11.8 ± 1.6#	11.5 ± 1.1#
	**HFD/WL**	9.9 ± 0.7	10.0 ± 0.8	9.9 ± 0.7
	**HFD/WL-S**	10.8 ± 1.4	10.9 ± 1.0	10.8 ± 1.2#

Right and left quadriceps, gastrocnemius, and soleus muscle mass (mg) for control (Chow, n:10), high fat diet (HFD, n:9), high fat diet secretome treated (HFD-S, n:9), weight loss (HFD/WL, n:9), and weight loss secretome treated (HFD/WL-S, n:10) mice. Right limbs received secretome injections throughout the experiment. All data collect at the 4-week timepoint. Analyzed via one way ANOVA, # = significant different from Chow, * = significant difference between HFD and HFD-S or HFD/WL and HFD/WL-S groups, respectively * <0.05.

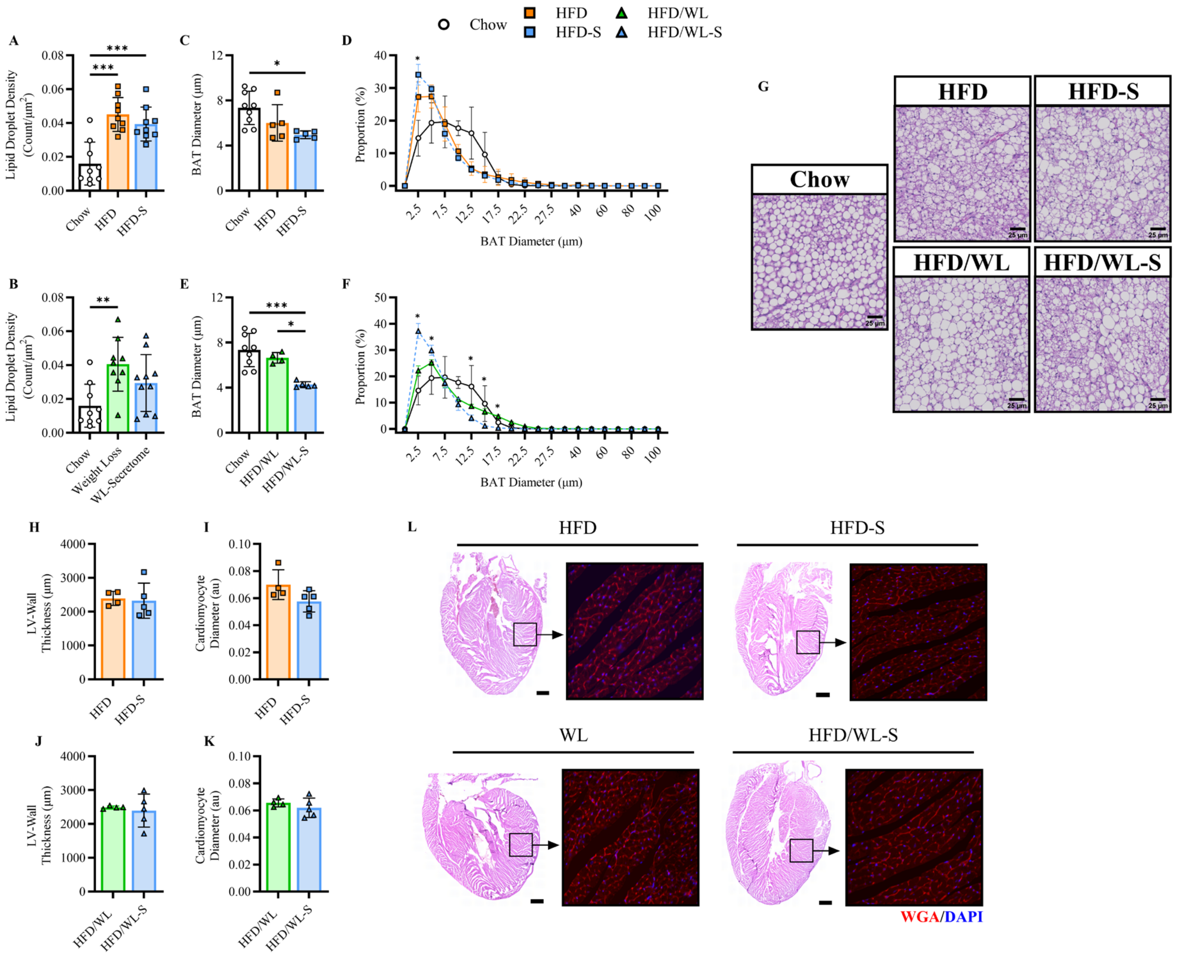

Figure S4. Additional Liver, Brown Adipose, and Heart Histology. A: Liver lipid droplet density (count/µm^2) for high fat diet (HFD, n:9) and high fat diet secretome treated (HFD-S, n:9) compared to chow control (Chow, n:9) mice. **C** and **D**: Average adipocyte diameter (µm) and size distribution (%) in brown adipose tissue (BAT) from HFD (n:9), HFD-S (n:5), and Chow mice (n:5). **B**: Liver lipid droplet density for weight loss (HFD/WL, n:9), and weight loss secretome treated (HFD/WL-S, n:10) compared to Chow mice. **E** and **F**: Average adipocyte diameter and size distribution in BAT from HFD/WL (n:4), HFD/WL-S (n:5), and Chow mice. G: Representative image for BAT H&E staining (scale: 25 µm). **H-K**: Left ventricular wall (LV-Wall) thickness (µm) and cardiomyocyte diameter (au) for HFD (n:4), HFD-S (n:5), HFD/WL (n:4), and HFD/WL-S (n:5). L: Representative image of whole heart histology (H&E) and cardiomyocytes from the left ventricle (WGA: red, DAPI: blue). All data collect at the 4-week timepoint. Analyzed using t-tests or one-way ANOVAs. * = significant difference between groups, * <0.05, ** <0.01, *** <0.001.

Supplementary Methods – Tissue Histology

Paraffin sections were dewaxed by xylene submersion (2 x 5-10 min) and ethanol (100%, 95%, 70%, 50%, 0%) rehydration (3 min each). For H&E staining livers and fat pads were incubated in Mayer's hematoxylin for 3-10 min, rinsed in H2O and 0.5% acetic acid, incubated in Eosin for 30 s, rinsed in H2O, dehydrated in ethanol (90%, 95%, 100%), dipped in xylene, then mounted and coverslipped. To stain capillary density, muscle sections were fixed in acetone for 10 min at -20°C, washed 3x in PBS, then blocked in H2O2 (3%) for 7 min. Sections were washed and blocked in horse serum (2.5%) for 1 hour and washed. Primary antibody incubation (CD31 1:100 (550274, BD Biosciences, Franklin Lakes, NJ, USA) was performed overnight at 4°C. Following 3x washes, slides were incubated in secondary antibodies (CD31, 1:250, A10522, Thermo Fisher Scientific, Waltham, MA, USA) for 1 hour at room temperature followed by 10 min in wheat germ agglutin (WGA, 1:200, W32466, Thermo Fisher Scientific) and DAPI (1:10,000, D3571, Invitrogen, Waltham, MA, USA), 3x washes, then mounted (Vectashield H-1000, Vector Laboratories, Newark, CA, USA) and coverslipped. Hearts were fixed in paraformaldehyde (4%) for 10 min, washed 3x, then stained with H&E or WGA and DAPI as described. Sirius red staining of liver and muscle included 60 min fixation in Bouin's Solution (HT10132, Sigma-Aldrich, St. Lous, MO, USA) at 56°C, rinsed 3x in H2O, then incubated in Sirius Red solution (ab246832, Abcam, Cambridge, UK) for 1 hour on a rocker at room temperature. Slides were washed 3x in acetic acid (0.5%), rinsed and dehydrated in ethanol, dipped in xylene, then mounted (Cytoseal XYL, Fisher Scientific) and coverslipped. Brightfield and fluorescent images were obtained at 10-20X using an Axio scan.z1 (Carl Zeiss, Oberkocken, DE) or EVOS FL microscope (Thermo Fisher Scientific). Myofiber CSA and capillarity density were analyzed using MuscleJ 2.0 and manually analysis [49], fat pad adipocyte size was using the Adiposoft plugin [50], and liver adiposity in Fiji by semi-automated thresholding and particle analysis.
